# Efficacy and Safety of Docetaxel and Sodium Cantharidinate Combination *vs.* Either Agent Alone as Second-Line Treatment for Advanced/Metastatic NSCLC With Wild-Type or Unknown EGFR Status: An Open-Label, Randomized Controlled, Prospective, Multi-Center Phase III Trial (Cando-L1)

**DOI:** 10.3389/fonc.2021.769037

**Published:** 2021-12-14

**Authors:** Lin Wu, Chao Deng, Hui Zhang, Jie Weng, Youhua Wu, Shan Zeng, Tiegang Tang, Peiguo Cao, Bo Qiu, Li Zhang, Huaxin Duan, Bing Zhang, Dong Zhang, Taotao Zhang, Chunhong Hu

**Affiliations:** ^1^ Department of Oncology, The Second Xiangya Hospital of Central South University, Changsha, China; ^2^ Department of Thoracic Medicine, Hunan Cancer Hospital, Changsha, China; ^3^ Department of Oncology, The Central Hospital of Shaoyang, Shaoyang, China; ^4^ Department of Oncology, The First People’s Hospital of Yueyang, Yueyang, China; ^5^ Department of Oncology, The First Affiliated Hospital of South China University, Hengyang, China; ^6^ Department of Oncology, The Xiangya Hospital of Central South University, Changsha, China; ^7^ Department of Oncology, The Central Hospital of Xiangtan, Xiangtan, China; ^8^ Department of Oncology, The Third Xiangya Hospital of Central South University, Changsha, China; ^9^ Department of Oncology, The Central Hospital of Zhuzhou, Zhuzhou, China; ^10^ Department of Oncology, Peking Union Medical College Hospital, Beijing, China; ^11^ Department of Oncology, Hunan Provincial People’s Hospital and The First Affiliated Hospital of Hunan Normal University, Changsha, China; ^12^ Department of Oncology, The Central Hospital of Yiyang, Yiyang, China; ^13^ Department of Oncology, Chinese PLA General Hospital, Beijing, China; ^14^ Guizhou Jinqiao Pharmaceutical Co., Ltd., Guiyang, China

**Keywords:** non-small cell lung cancer, sodium cantharidinate, docetaxel (DOC), combination, efficacy and safety

## Abstract

Second-line treatment options for advanced/metastatic non-small cell lung cancer (NSCLC) patients are limited. We aimed to evaluate the efficacy and safety of docetaxel/sodium cantharidinate combination *vs*. either agent alone as second-line treatment for advanced/metastatic NSCLC patients with wild-type or unknown EGFR status. A randomized, open-label, phase III study was performed at 12 institutions. Patients with failure of first-line platinum regimens were randomized to receive either single-agent sodium canthari*vs*dinate (SCA) or single-agent docetaxel (DOX) or docetaxel/sodium cantharidinate combination (CON). The primary endpoints were centrally confirmed progression-free survival (PFS) and overall survival (OS). The secondary endpoints were objective response rate (ORR), disease control rate (DCR), quality of life (QoL) and toxicity. A total of 148 patients were enrolled in our study between October 2016 and March 2020. After a median follow-up time of 8.02 months, no significant difference was observed among the three groups in ORR (SCA *vs*. DOX *vs*. CON: 6.00% *vs*. 8.33% *vs*. 10.00%, respectively; p=0.814) and DCR (74.00% *vs*. 52.00% *vs*. 62.50%, respectively; p=0.080). In additional, the mOS was significantly higher in the CON group, compared with the single-agent groups (7.27 *vs*. 5.03 *vs*. 9.83 months, respectively; p=0.035), while no significant differences were observed in terms of PFS (2.7 *vs*. 2.9 *vs*. 3.1 months, respectively; p=0.740). There was no significant difference in the baseline QoL scores between the three groups (p>0.05); after treatment, life quality in SCA and CON group was significantly better than that in the DOX group (p<0.05). Furthermore, the incidence of adverse events (AEs) in the SCA group was significantly lower (46.00 *vs*. 79.17 *vs*. 25.00%, respectively; p=0.038) and the incidence of grade ≥3 AEs was also significantly lower in the SCA group compared with the DOX and CON groups (10.00 *vs*. 82.00 *vs*. 30.00%, respectively; p=0.042). Single-agent SCA and single-agent DOX has similar therapeutic efficacy in the second-line treatment of advanced/metastatic NSCLC with wild-type or unknown EGFR status, but single-agent SCA has fewer AEs and better QoL. Also, SCA plus DOX can significantly improve OS and exerted a significant synergistic effect, with good safety and tolerance profile.

## Introduction

Lung cancer is the most common malignant tumor and one of the major causes of cancer-related mortality worldwide (1). Non-small cell lung cancer (NSCLC) accounts for 80% of all lung cancer cases (2), with >70% of the patients already having advanced or metastatic lesions when diagnosed and having missed the opportunity for radical surgery (3, 4). Comprehensive treatment based on systemic therapy is the main principle for advanced/metastatic NSCLC. For patients with mutations of driver genes, such as EGFR, targeted therapy has become the standard choice for first- and second-line treatment (5). However, EGFR-sensitive mutations are more common among non-smoking, female lung adenocarcinoma patients. Among Chinese NSCLC patients who are heavy smokers, the EGFR gene is often wild-type, and sensitive mutations in other driver genes, such as EML4-ALK gene rearrangements, are also rare (6). For a large proportion of such patients, chemotherapy is the only option for first- and second-line treatment (7). Platinum-based chemotherapy is currently considered as the standard first-line treatment, but almost all patients for whom the initial therapy was effective develop disease progression within 3-4 months and require second-line treatment (8).

Second-line chemotherapy includes docetaxel (DOX) and pemetrexed. For patients with no driver gene mutations or previous first-line treatment with pemetrexed, DOX is the main second-line treatment option, but its efficacy is limited, with a disease control rate of 53% and a median survival time of 5.5-7.9 months. In addition, several patients are unable to complete treatment due to adverse events (8). In recent years, Immunotherapy (PD-1/L1) combined with chemotherapy has achieved success in the first/second-line treatment of NSCLC without driver mutations (9, 10). Studies have shown that (11) the ORR of pembrolizumab combined with DOX in the second-line treatment of advanced NSCLC without driver mutations is 42.5% (*vs*. 15.8%), and the median progression-free survival is 9.5 months (*vs*. 4.1 months), which is significantly better than single-agent docetaxel. However, In terms of safety, 23% *vs* 5% of patients experienced pneumonitis in the pembrolizumab plus DOX and DOX arms. It is currently believed that patients with second-line treatment have poor lung function due to previous treatment and disease progression, and immunotherapy combined with chemotherapy has greatly increased the risk of pneumonia. Therefore, the second-line treatment of NSCLC with immunization combined with chemotherapy still needs more research to determine its safety, and it is necessary to select suitable patients to benefit. Overall, Second-line treatment options for advanced/metastatic wild-type or unknown EGFR status NSCLC patients with failure of first-line platinum regimens are limited.

Mylabris is a coleopteran insect (12). As early as two thousand years ago, there was a record of Mylabris treating tumors in China. Cantharidin (molecular formula, C_10_H_12_O_4_) is the effective component extracted from the body of *Mylabris* (13). Sodium cantharidinate (SCA) is a semi-synthetic derivative of cantharidin, the antitumor efficacy of SCA is significantly better and its toxicity is significantly lower compared with that of cantharidin (14). The anticancer effects of SCA are mainly mediated by restricting cell cycle progression from the S phase to G/M phase in cancer cells, thereby inhibiting cell proliferation and inducing cell apoptosis, similar to the action of cytotoxic drugs (15). Clinical studies have shown that SCA combined with hepatic artery perfusion have achieved significant efficacy in the treatment of patients with hepatocellular carcinoma (HCC) (16), and SCA injection was also approved by the China Food and Drug Administration in 2013 for HCC (17). Recent clinical observations have found that SCA combined with chemotherapy exerted a significant synergistic effect in the treatment of esophageal, gastric, pancreatic and cervical cancer, osteosarcoma and other solid tumors, without increasing the incidence of adverse events (18–21). In 2014, Wang et al. (22) conducted a clinical study found that the clinical efficacy of SCA combined with GP regimen in the treatment of advanced NSCLC was better than the conventional GP regimen. However, there is no large-scale phase III clinical trial of SCA in patients with advanced NSCLC, to the best of our knowledge.

In order to explore the efficacy and safety of SCA as a single agent or combined with chemotherapy in the second-line treatment of patients with advanced NSCLC, we designed and carried out a randomized, open-label, national multi-center phase III clinical trial, including selected patients with advanced/metastatic NSCLC with wild-type or unknown EGFR status following failure of first-line platinum regimens. The patients were randomized to receive either DOX/SCA combination (CON) or single-agent DOX or single-agent SCA until disease progression or the development of intolerable adverse events, and the clinical efficacy and safety of the three treatment options were compared. The findings of the present study may provide important clinical evidence for the application of SCA as a single agent or combined with chemotherapy in the second-line treatment of patients with advanced NSCLC.

## Patients and Methods

### Patients

This study is a randomized, open-label, prospective, national multi-center phase III clinical trial led by the Cancer Center of the Second Xiangya Hospital of Central South University. The subjects were recruited from 12 research centers across the country, including the Second Xiangya Hospital of Central South University, Hunan Cancer Hospital and Peking Union Medical College Hospital. The criteria for entry and discharge of the subjects were as follows: Patient eligibility criteria included i) Age 18-75 years; ii) Eastern Cooperative Oncology Group (ECOG) performance status (PS) score 0-1; iii) Advanced or metastatic NSCLC confirmed by histology/cytology; iv) patients with failure of first-line platinum-based regimens (except for docetaxel-based regimens) who had not received targeted therapies, such as EGFR-TKIs; v) wild-type or unknown EGFR status; vi) patients with at least one measurable lesion according to the standard Response Evaluation Criteria in Solid Tumors (RECIST)1.1 (21); vii) patients who had not received surgery or radiotherapy within 4 weeks, and who had completely healed surgical incisions and/or recovered fully from the side effects of radiotherapy; viii) life expectancy of ≥3 months; and ix) adequate organ and bone marrow function. Patients with symptomatic brain metastases, patients with severe allergies to docetaxel or sodium cantharidinate, patients receiving other systemic antitumor therapy, patients with severe active infection, pregnant or lactating women, as well as alcohol or drug abusers, were excluded from the present study.

This study was approved by the medical ethics committees of the participating centers and registered in the Chinese Clinical Trial Database (registration no. **ChiCTR-IPR-16009159**) (22). The research protocol strictly complied with the principles outlined in the Declaration of Helsinki (23), and was carried out in accordance with the Provisions for Drug Registration and Good Clinical Practice (GCP) issued by the State Food and Drug Administration (SFDA). All subjects signed written informed consent forms.

### Study Design

This was a randomized, open-label, prospective, national multi-center phase III study, grouped through the online registration system. Patients were enrolled between October 2016 and March 2020. SCA injection (0.25 mg/5 mL) was developed and provided by Jinqiao Pharmaceutical Co., Ltd. (Guizhou, China), and DOX (20 mg/0.5 mL) was purchased from Qilu Pharmaceutical Co., Ltd. (Shandong, China). Patients were randomized to either the CON group (DOX 75 mg/m^2^, d1; sodium cantharidinate 0.5 mg, d1-14; repeated every 21 days), or the DOX group (single-agent DOX 75 mg/m^2^, d1; repeated every 21 days), or the SCA group (single-agent sodium cantharidinate 0.5 mg, d1-14; repeated every 21 days), until disease progression or the development of intolerable adverse events. During the course of treatment, if grade ≥3 hematological or grade ≥2 non-hematological toxicities develop, a second dose reduction of docetaxel is allowed. In the 75 mg/m^2^ dose group, the first dose will be adjusted to 60 mg/m^2^, and the second dose will be adjusted to 50 mg/m^2^. Once the dose is adjusted, the study will be completed according to the reduced dose. If grade 3/4 adverse reactions according to the National Cancer Institute Common Terminology Criteria for Adverse Events (NCI_CTCAE) 4.03 still occur after two dose adjustments (24), treatment must be discontinued. For sodium cantharidinate, dose adjustment is not allowed, but dose interruption is allowed, and the maximum interruption time does not exceed 5 days.

### Effectiveness and Safety Assessment

The primary endpoints were centrally confirmed progression-free survival (PFS) and overall survival (OS). Secondary endpoints included objective response rate (ORR), disease control rate (DCR), quality of life (QoL) and toxicity. The standard RECIST1.1 were used to evaluate the objective curative effect on tumors, which were divided into complete response (CR), partial response (PR), stable disease (SD) and progressive disease (PD). PFS is defined as the time between when subjects are randomized to the group and first confirmation of disease progression or death from any cause (whichever occurs first); the follow-up time for PFS is 12 months. OS refers to the time from random enrollment to death from any cause. Patients whose death date is not recorded in the clinical database are censored at the last known alive date; the follow-up time for OS is 24 months. ORR is defined as the percentage of CR and PR cases and DCR is defined as the percentage of CR, PR and SD cases among patients with evaluable efficacy following by RECIST version 1.1. The adverse events (AEs) of the subjects were evaluated according to the standard NCI_CTCAE version 4.03, and the clinical characteristics, severity, time of occurrence, duration, treatment methods and prognosis were recorded. The QoL were evaluated by Functional Assessment of Cancer Therapy-Lung (FACT-L) every 3 weeks.

### Statistical Analysis

SPSS Statistics, version 24.0 (IBM Corp) and GraphPad Prism (version 8.0) were used to perform the statistical analysis. Continuous variables were presented as the mean and standard deviation (SD) or median and interquartile range (IQR) and compared using the Wilcoxon rank-sum test or Kruskal-Wallis test. Categorical variables were described by frequencies and percentages and compared by Fisher exact and χ^2^ tests. The Kaplan-Meier method and log-rank test were used to evaluate PFS and OS durations. All statistical tests were two-sided, α=0.05, and *P*<0.05 was considered statistically significant.

## Results

### Patient Baseline Characteristics

**Figure 1:**
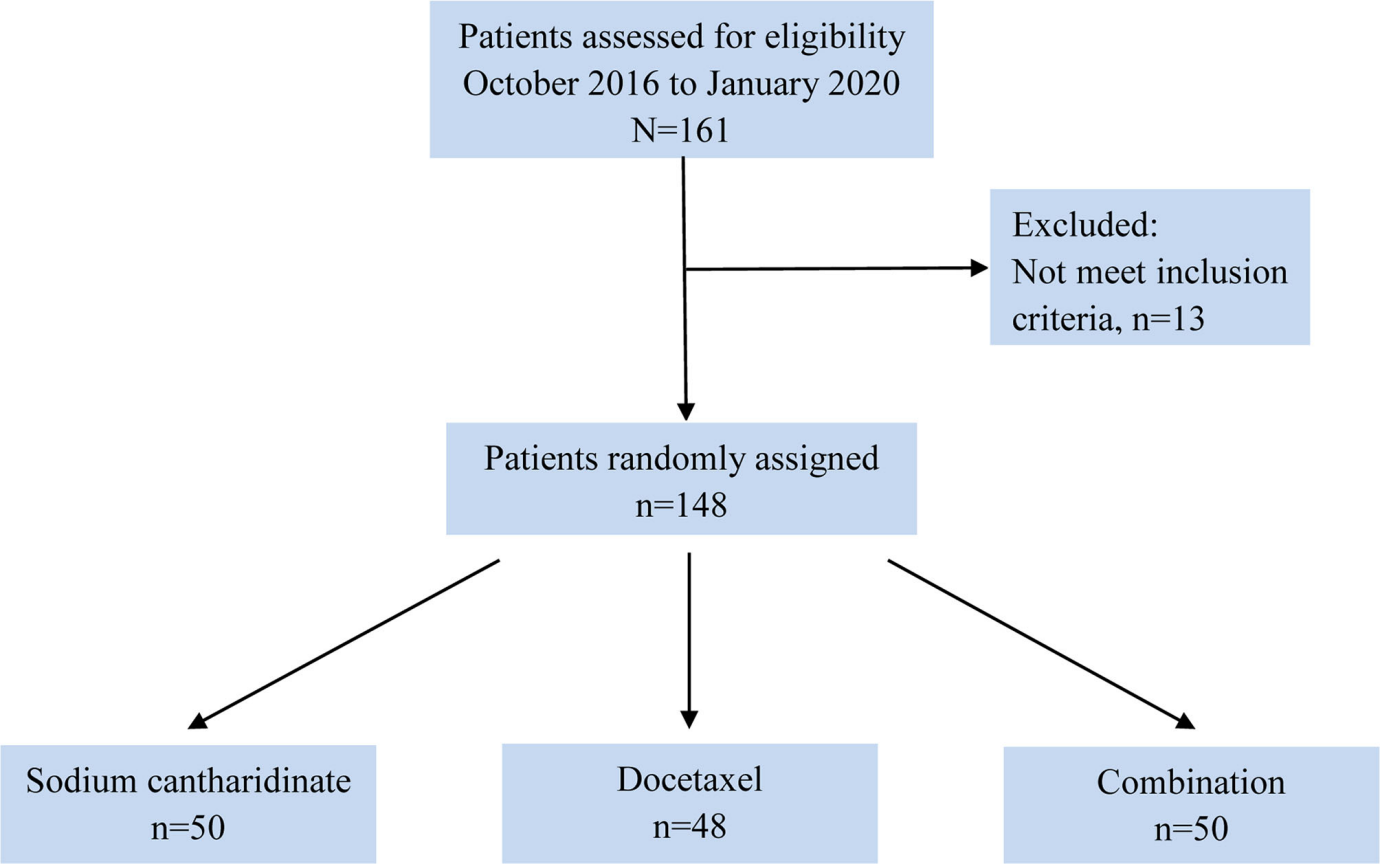
Flow diagram of patient selection.

A total of 148 patients were enrolled in our study between October 2016 and March 2020 at 12 institutions (**Supplementary Table 1**). Of the 148 eligible patients, 50 (33.78%) were assigned to the SCA group, 48 (32.44%) were assigned to the DOX group and 50 (33.78%) were assigned to the CON group (**Figure 1**). The median age was 51 years (IQR: 44-61), and 124 patients (83.78%) were male. All patients had stage IV disease, adenocarcinoma and squamous cell carcinoma were the most common histological types (accounting for 40.54 and 56.08%, respectively), and EGFR wild-type patients accounted for 49.32% of the cases (**Supplementary Table 2**). There were no significant differences among the three groups in terms of age (p=0.928), sex (p=0.888), ECOG PS score (p=0.957), histological type (p=0.799), Charlson Comorbidity Index (CCI) (p=0.916), or EGFR status (p=0.735) (**Table 1**).

**Table 1:** Patients’ baseline demographic and clinical characteristics in each group (N=148).

Characteristic	SCA (n=50)	DOX (n=48)	CON (n=50)	p
Median age at enrollment – years (IQR), n=148	62 (39-69)	51 (46-59)	56 (52-63)	0.928
Male sex– no. (%)	41	40	43	0.888
Smoking– no. (%)	35	29	40	0.109
Median ECGO at enrollment – points (IQR), n=148	1 (1-1)	1 (1-1)	1 (1-1)	0.957
Median Charlson Comorbidity Index (CCI) at enrollment, points. (IQR), n=148	8 (7-8)	8 (7-8)	7 (7-8)	0.916
Cancer stage IV– no. (%)	50 (100)	48 (100)	50 (100)	1.00
Histological type– no. (%)				0.799
Adenocarcinoma	23	17	20	
Squamous cell carcinoma	25	29	29	
Others				
EGFR status– no. (%)				0.735
Wild-type	24	26	23	
Unknown	26	22	27	
Median Treatment cycle, (IQR)	2 (1-3)	2 (1-3.75)	2.5 (2-4)	0.092

*IQR, denotes interquartile range; SD, Standard Deviation; CCI, Charlson Comorbidity Index.

### Clinical Efficacy Analysis

In our cohort, 12 patients achieved PR after treatment, 81 patients achieved SD, whereas no patients achieved CR; the overall ORR was 8.11%, and the overall DCR was 62.84% (**Table 2**). The ORRs of the three groups were 6.00, 8.33 and 10.00%, respectively, with no statistically significant differences (**Table 2**; p=0.469). The DCR of the CON group was 74.00%, which was higher compared with that of the SCA group (52.00%) and the DOX group (62.50%), but without statistical significance (**Table 2**; p=0.080). There was also no statistical significance between the SCA and DOX groups (p=0.437).

**Table 2:** Comparison of response rate between three groups (N=148).

Characteristic	Total (N=148)	SCA (n=50)	DOX (n=48)	CON (n=50)	p
PR– no. (%)	12 (8.11)	3 (6.00)	4 (8.33)	5 (10.00)	–
SD– no. (%)	81 (54.73)	23 (46.00)	26 (54.17)	32 (64.00)	–
ORR– no. (%)	12 (8.11)	3 (6.00)	4 (8.33)	5 (10.00)	0.814
DCR– no. (%)	93 (62.84)	26 (52.00)	30 (62.50)	37 (74.00)	0.080

*PR, partial response; SD, stable disease; ORR, objective response rate; DCR, disease control rate.

**Figure 2:**
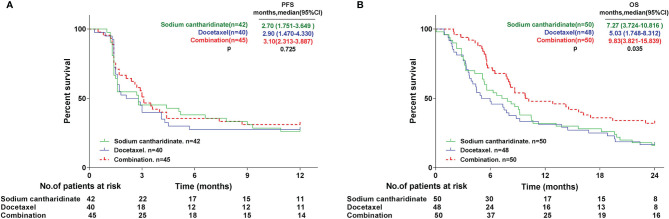
Kaplan-Meier curves for **(A)** progression-free survival (PFS) and **(B)** overall survival (OS).

The primary endpoints were centrally confirmed PFS and OS. All subjects were followed up for 12 months for PFS. A total of 127 patients were included in the analysis set. The results revealed that the median PFS (mPFS) of the SCA, DOX and CON groups was 2.7 (95% CI: 1.751-3.649), 2.9 (95% CI: 1.470-4.330) and 3.1 (95% CI: 2.313-3.887) months, respectively. The mPFS of the CON group was slightly higher compared with that of the SCA and DOX group. The mPFS was similar between the SCA and CON groups, and no statistically significant difference in mPFS was observed among the three groups (**Figure 2A**; p=0.725). All subjects were followed up for 24 months for OS. All subjects were included in the analysis set. A total of 116 patients succumbed to tumor progression. The results revealed that the median OS (mOS) of the SCA, DOX and CON groups was 7.27 (95% CI: 3.724-10.816), 5.03 (95% CI: 1.748-8.312) and 9.83 (95% CI: 3.821-15.839) months, respectively. mOS was significantly higher in the CON group, compared with that in the SCA and DOX groups (**Figure 2B**, p=0.035). Furthermore, the mOS of the SCA group was higher compared with that of the DOX group, but the difference was not statistically significant (p=0.711). Subgroup analysis shows that there were no statistically significant difference in mOS was observed among histological type (Adenocarcinoma *vs*. Squamous cell carcinoma, **Supplementary Figure 1A**, p=0.558) and EGFR status (Wild-type *vs*. Unknown, **Supplementary Figure 1B**, p=0.163)

### QoL Assessment

In our study, the QoL was evaluated by Functional Assessment of Cancer Therapy- Lung (FACT-L) every 3 weeks. The FACT-L scale includes four dimensions: FACT physical well-being, FACT emotional well-being, FACT social well-being, and FACT functional well-being. The FACT physical/emotional score are inversely proportional to QoL, and FACT social/functional status score are directly proportional to QoL. The results demonstrated that there was no significant difference in the baseline QoL scores between the three groups (**Table 3**; p>0.05); after treatment, the QoL in the SCA and CON group were significantly improved (**Table 3**; p<0.05); while the physical/emotional status of DOX group were better than before treatment (**Table 3**; p<0.05), but the social/functional status were decreased (**Table 3**; p<0.05).

**Table 3:** FACT-L score in three groups before and after treatment.

Variable	Group	Baseline	3 weeks	6 weeks	9 weeks
FACT physical	SCA	12.84 (5.85)	9.89 (4.39)	8.13 (5.40)	8.12 (5.97)
	DOX	12.07 (4.28)	9.55 (4.53)	9.50 (6.29)	9.10 (4.02)
	CON	11.33 (4.24)	9.40 (5.69)	7.22 (3.99)	8.40 (4.80)
FACT emotional	SCA	7.82 (5.03)	7.22 (4.62)	7.28 (3.95)	6.88 (4.20)
	DOX	7.61 (3.71)	6.32 (2.93)	7.17 (4.18)	7.78 (3.86)
	CON	7.02 (3.79)	7.30 (3.51)	6.17 (2.54)	6.53 (3.28)
FACT social	SCA	19.42 (4.41)	18.19 (5.79)	20.38 (5.94)	22.25 (3.53)
	DOX	19.32 (5.80)	17.35 (5.53)	19.83 (7.29)	18.60 (5.28)
	CON	20.15 (5.42)	19.75 4.66)	21.06 (5.99)	21.40 (3.52)
FACT functional	SCA	12.85 (5.85)	12.78 (5.87)	14.87 (4.96)	15.55 (6.44)
	DOX	12.27 (6.43)	11.03 (6.12)	11.67 (6.88)	10.60 (6.04)
	CON	12.65 (6.44)	14.88 (7.04)	14.38 (7.38)	17.46 (7.12)

### Adverse Events

**Figure 3:**
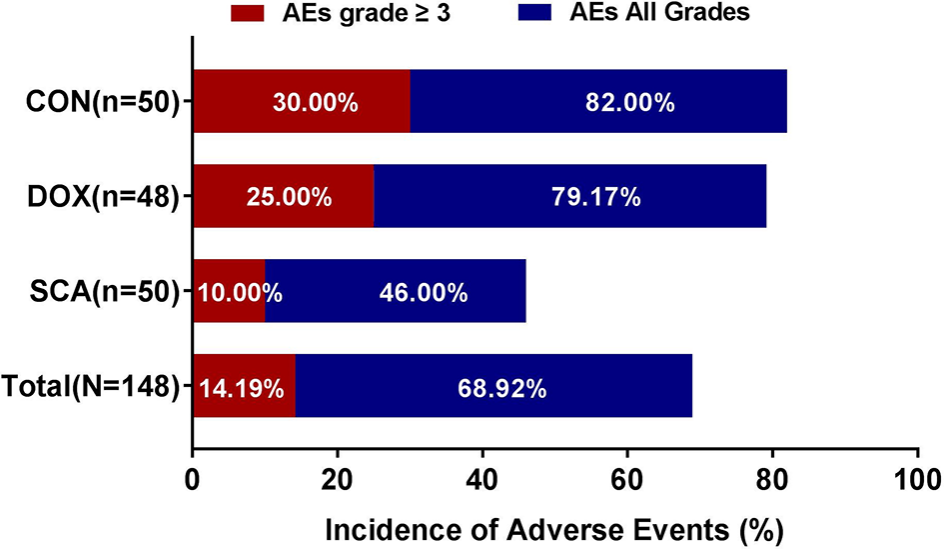
Incidence of Adverse Events in each group (N=148).

Among the 148 subjects, 102 (68.92%) developed AEs and 32 (14.19%) had grade ≥3 AEs; no treatment-related fatalities were recorded. The incidence of AEs (**Figure 3**; SCA *vs*. DOX *vs*. CON: 46.00% *vs*. 79.17% *vs*.82.00%, respectively, p=0.038) and grade ≥3 AEs (**Figure 3**; SCA *vs*. DOX *vs*. CON: 10.00% *vs*. 25.00% *vs*. 30.00%, respectively, p=0.042) in the SCA group were significantly lower compared with those in the DOX group and the CON group. There was no statistically significant difference in the rates of AEs and grade ≥3 AEs between the DOX and CON groups (**Figure 3**; p=0.745 and p=0.548, respectively). The major hematological AEs were neutropenia and decreased hemoglobin concentration and platelet count (**Supplementary Table 3**). The major non-hematological AEs were asthenia, fatigue, lethargy, nausea/vomiting, increase in ALT and AST, constipation, abdominal pain, diarrhea, increase in creatinine levels and muscle pain (**Supplementary Table 3**).

## Discussion

*Mylabris* is a coleopteran insect (12). The earliest Chinese medicine monograph “Sheng Nong’s herbal classic” has recorded the use of cantharides for therapeutic purposes. It is cold and pungent in nature, and has significant toxicity; its known therapeutic properties are mainly to disperse swelling, activate blood circulation and remove stasis (Traditional Chinese Medicine terminology). Cantharidin (molecular formula, C_10_H_12_O_4_) is the effective component extracted from the body of *Mylabris* (15). The antitumor efficacy of cantharidin has received extensive attention in recent years. Studies (25) have shown that cantharidin can specifically inhibit the activity of protein phosphatase, regulate tumor cell autophagy, and participate in the regulation of tumor cell proliferation and apoptosis. However, the natural toxicity of cantharidin limits its clinical use.

SCA is a semi-synthetic derivative of cantharidin, which is chemically synthesized from cantharidin and sodium hydroxide (molecular formula, C_10_H_12_Na_2_O_5_); the antitumor efficacy of SCA is significantly better and its toxicity is significantly lower compared with that of cantharidin (26–28). The anticancer effects of SCA are mainly mediated by restricting cell cycle progression from the S phase to G/M phase in cancer cells, thereby inhibiting cell proliferation and inducing cell apoptosis, similar to the action of cytotoxic drugs (15). It has been demonstrated that (29) SCA can also enhance immune function by promoting the proliferation of T lymphocytes and secreting the cytokine interleukin-2. It has also been shown that (30) SCA inhibits the secretion of interleukin-8, downregulates the protein expression of VEGF and MMP-9, inhibits the formation of new blood vessels, inhibits tumor cell adhesion, thereby inhibiting tumor invasion and metastasis. More studies (29) have shown that SCA can shorten the bone marrow maturation and release time of leukocytes, and promote the differentiation of hematopoietic stem cells into granulocyte/monocyte progenitor cells, while increasing white blood cell count, thereby reducing the hematopoietic system toxicity of chemoradiotherapy.

Clinical studies have shown that SCA combined with hepatic artery perfusion have achieved significant efficacy in the treatment of patients with hepatocellular carcinoma (HCC) (16), and SCA injection was also approved by the China Food and Drug Administration in 2013 for HCC (17). Recent clinical (18–21, 31–34) observations have found that SCA combined with chemotherapy exerted a significant synergistic effect in the treatment of esophageal, gastric, pancreatic and cervical cancer, osteosarcoma and other solid tumors, without increasing the incidence of adverse events. In 2014, Wang et al. (22) conducted a clinical study to observe the clinical outcomes of the SCA combined with GP regimen in the treatment of middle-to-late-stage NSCLC. A total of 86 NSCLC patients were enrolled, of whom 45 patients were randomized to the SCA + GP combination group, and 41 patients received the GP regimen alone. The effectiveness rate of the combination and GP groups was 57.8 and 36.6%, respectively (p<0.05); HRQoL in the combination group was significantly higher compared with that in the GP group (p<0.05); the occurrence rate of toxic/adverse effects was significantly lower in the combination group (p<0.05). However, no survival-related data were collected and analyzed. Zheng et al. (35) further conducted a meta-analysis of 38 trials involving 2,845 patients in China, and found that SCA could increase the ORR (1.52 [1.40-1.66]), DCR (1.20 [1.16-1.25]) and QoL (1.76 [1.56-1.98]), but not the 1- and 2-year OS rate (1.16 [0.91-1.47] and 1.21 [0.51-2.91], respectively). Subgroup analysis revealed that SCA was associated with a lower risk of nausea/vomiting, gastrointestinal reactions, neutropenia and thrombocytopenia compared with that of chemotherapy alone.

In current clinical practice, for patients with no driver gene mutations, DOX is the main second-line treatment option, but DOX not only has limited efficacy, but also several patients are unable to complete treatment due to adverse events (8). Although immunotherapy (PD-1/L1) combined with DOX significantly improves the therapeutic effect, but patients with second-line treatment have poor lung function, and immunotherapy combined with chemotherapy can greatly increase the risk of pneumonia, which restricts second-line use (9). Overall, Second-line treatment options for advanced/metastatic wild-type or unknown EGFR status NSCLC patients with failure of first-line platinum regimens are limited. From previous studies, SCA is expected to become an important choice for second-line treatment of advanced/metastatic NSCLC with wild-type or unknown EGFR status. However, there is no large-scale phase III clinical trial of SCA in patients with advanced NSCLC, to the best of our knowledge. In order to explore the efficacy and safety of SCA as a single agent or combined with chemotherapy in the second-line treatment of patients with advanced NSCLC, we designed and carried out a randomized, open-label, national multi-center phase III clinical trial for the first time.

Through strict entry and exclusion criteria, 148 advanced/metastatic NSCLC patients with wild-type or unknown EGFR status were enrolled in our study, and randomly assigned to CON group (n=50) or DOX group (n=48) or SCA group (n=50) until disease progression or the development of intolerable adverse events. In our cohorts, there were no significant differences among the three groups in terms of age, gender, ECOG, histological type, CCIs, EGFR status. Among the 148 subjects investigated in the present study, all patients had at least one evaluable CT scan. Statistical results demonstrated that the overall ORR was 8.11%, and the overall DCR was 62.84%. The ORRs of the three groups were 6.00, 8.33 and 10.00%, respectively, with no statistically significant differences. The ORRs of the three groups were 52.00, 62.50 and 74.00%, respectively, although there was no statistically significant difference among the three groups, the DCR of the CON group was found to be significantly higher compared with that of the single-agent groups. The ORR and DCR of docetaxel in our study were 8.33% and 62.5%, which were basically in line with the results of previous clinical studies (8, 9, 36, 37). Our study found that single-agent SCA and single-agent DOX has similar ORR and DCR, the clinical treatment effect is equivalent; and SCA combined with DOX can improve the DCR to a certain extent, with a synergistic effect.

We further performed statistical analysis on the primary endpoints PFS and OS. All subjects were followed up for 12 months for PFS. A total of 127 patients were included in the analysis set. The statistical results revealed that the mPFS of the three groups were 2.70, 2.90 and 3.10 months; there was no significant difference in mPFS among the three groups. It is worth noting that one subject (NO.14001) in the SCA group completed the 22-cycle dosing trial, with a PFS of 19 months. All subjects were followed up for 24 months for OS, and all subjects were included in the analysis set. A total of 116 patients succumbed to tumor progression. It was found that the mOS of the three groups were 7.27, 5.03 and 9.83 months, respectively. The mOS of the CON were significantly higher compared with that of the two single-agent groups. Interestingly, the mOS of the SCA group were higher compared with that of the DOX group, although the difference was not statistically significant. These results indicate that SCA alone has achieved PFS and OS benefits that are not inferior to single-agent DOX in the second-line treatment of advanced/metastatic NSCLC with wild-type or unknown EGFR status. And, although SCA plus DOX did not benefit from PFS, the mOS of the CON group was significantly prolonged by 4.8 months compared with single-agent DOX, and OS was significantly benefited in CON group.

In terms of QoL, there was no significant difference in the baseline QoL (38) scores between the three groups; after treatment, the QoL in the SCA and CON group were significantly improved; while the physical/emotional status of DOX group were better than before treatment, but the social/functional status were decreased. These results indicate that compared with single-agent DOX group, single-agent SCA or combination therapy can effectively improve the QoL of patients. Also, in terms of safety, a total of 148 subjects in the three groups were evaluated for the presence of AEs, among whom 68.92% had AEs, 14.19% had grade ≥3 TEAEs, whereas no fatalities due to serious adverse reactions were recorded. Among them, the incidence of AEs of three groups were 46.00%, 79.17%, and 82.00%, respectively; the incidence of ≥ grade 3 AEs were 10.00%, 25.00%, and 30.00%, respectively. These results indicate that compared with single-agent DOX, the incidence of AEs and ≥ Grade 3 AEs in the single-agent SCA group was significantly lower, especially hematology- related AEs, such as leukopenia/neutropenia, anemia and thrombocytopenia.; The SCA group mainly exhibited non-hematological AEs, such as weakness, fatigue, drowsiness, increased ALT and AST levels, and increased creatinine levels, and most of the AEs are transient or reversible, within the controllable range, and there were no unexpected AEs; most importantly, SCA plus DOX did not increase the incidence of AEs and grade ≥3 AEs. The safety and tolerability of single-agent SCA or combination therapy are both better.

There are several limitations in our study. Firstly, the sample size was too small and multiple group analyses may have been underpowered, and a larger sample size is needed to verify the OS benefit of patients (39). Secondly, as immunotherapy became the gold standard in all the approved guide lines (40), but the patients we enrolled in the first-line/second-line treatment did not receive immunotherapy; which may not fully reflect real-world clinical experience. Thirdly, our study is not a double-blind but open-label trial, subjective factors of researchers and participants will bring a certain bias (41). Fourthly, *in vitro* cell functional experiments were lacked in our study. Lastly, we believe that factors such as underlying disease activity, concomitant medications, and follow-up treatment in the patient population are unlikely to have confounded our data, so, our study did not analyze the effects of these factors on the efficacy and prognosis of patients, as it was beyond the scope of the current analyses.

## Conclusion

In summary, our prospective, randomized, open-label, national multi-center phase III clinical study demonstrated that single-agent SCA has similar ORR and DCR and PFS and OS benefits to single-agent DOX in the second-line treatment of advanced/metastatic NSCLC with wild-type or unknown EGFR status, with a lower risk of hepatotoxicity/gastrointestinal toxicity and better QoL. Therefore, SCA may be considered as an effective alternative treatment for patients with advanced/metastatic NSCLC with a poor general condition, who are unable to tolerate the side effects of chemotherapy. Furthermore, SCA plus DOX can significantly improve OS and exerted a significant synergistic effect, although it cannot improve PFS, ORR and DCR. Moreover, it does not increase the incidence of adverse reactions, and has a good safety and tolerance profile. To the best of our knowledge, this report is the first to provide valuable evidence on the efficacy and safety of SCA in the second-line treatment of patients with advanced NSCLC, which should affect clinical decision-making.

## Data Availability Statement

The original contributions presented in the study are included in the article/**Supplementary Material**. Further inquiries can be directed to the corresponding author.

## Ethics Statement 

This study was approved by the medical ethics committees of the participating centers and registered in the Chinese Clinical Trial Database (registration no. ChiCTR-IPR-16009159). The patients/participants provided their written informed consent to participate in this study. We highly appreciate the kind help and support from the Thoracic Oncology multidisciplinary team (MDT) of the Second Xiangya Hospital of Central South University.

## Author Contributions

CH was the senior author of this study, developed the concept, designed the study, interpreted the results, ensured that the accuracy and integrity of the data was preserved at all stages, agreed to be accountable for all aspects of this study, was in charge of the overall direction and planning of the study. LW and CD did the experimental studies, collected the data for the study, assessed to interpret the analysis, conducted biostatistical analysis and wrote the manuscript. HZ, JW, YW, SZ, TT, PC, BQ, LZ, HD, BZ, and DZ did the clinical studies, collected the data for the study, and revised critically the final version of the manuscript. All authors contributed to the article and approved the submitted version.

## Conflict of Interest

Author TZ was employed by company Guizhou Jinqiao Pharmaceutical Co., Ltd.

The remaining authors declare that the research was conducted in the absence of any commercial or financial relationships that could be construed as a potential conflict of interest.

## Publisher’s Note

All claims expressed in this article are solely those of the authors and do not necessarily represent those of their affiliated organizations, or those of the publisher, the editors and the reviewers. Any product that may be evaluated in this article, or claim that may be made by its manufacturer, is not guaranteed or endorsed by the publisher.

## Supplementary Material

Supplementary Figure 1 Kaplan-Meier curves for overall survival (OS) among **(A)** histological type and **(B)** EGFR status.

## References

[1] Duma N Santana-Davila R Molina JR. Non-Small Cell Lung Cancer: Epidemiology, Screening, Diagnosis, and Treatment. Mayo Clin Proc (2019) 94:1623–40. doi: 10.1016/j.mayocp.2019.01.013 31378236

[2] Siegel RL Miller KD Fuchs HE Jemal A. Cancer Statistics, 2021. CA Cancer J Clin (2021) 71:7–33. doi: 10.3322/caac.21654 33433946

[3] Nasim F Sabath BF Eapen GA. Lung Cancer. Med Clin North Am (2019) 103:463–73. doi: 10.1016/j.mcna.2018.12.006 30955514

[4] Yoneda K Imanishi N Ichiki Y Tanaka F. Treatment of Non-Small Cell Lung Cancer With EGFR-Mutations. J UOEH (2019) 41:153–63. doi: 10.7888/juoeh.41.153 31292359

[5] Herbst RS Morgensztern D Boshoff C. The Biology and Management of Non-Small-Cell-Lung-Cancer. Nature (2018) 553:446–54. doi: 10.1038/nature25183 29364287

[6] Herbst RS Garon EB Kim DW Cho BC Gervais R Perez-Gracia JL. 5-Year Survival Update From KEYNOTE-010: Pembrolizumab Versus Docetaxel for Previously Treated, Programmed Death Ligand 1-Positive Advanced Non-Small-Cell Lung Cancer. J Thorac Oncol (2021) 16:S1556–0864(21)02172-9. doi: 10.1016/j.jtho.2021.05.001 34048946

[7] Matsumoto K Tamiya A Matsuda Y Taniguchi Y Atagi S Kawachi H. Impact of Docetaxel Plus Ramucirumab on Metastatic Site in Previously Treated Patients With Non-Small Cell Lung Cancer: A Multicenter Retrospective Study. Transl Lung Cancer Res (2021) 10:1642–52. doi: 10.21037/tlcr-20-1263 PMC8107751 34012781

[8] Shepherd FA Dancey J Ramlau R Mattson K Gralla R O'Rourke M. Prospective Randomized Trial of Docetaxel Versus Best Supportive Care in Patients With Non-Small-Cell Lung Cancer Previously Treated With Platinum-Based Chemotherapy. J Clin Oncol (2000) 18:2095–103. doi: 10.1200/JCO.2000.18.10.2095 10811675

[9] Gubens MA Davies M. NCCN Guidelines Updates: New Immunotherapy Strategies for Improving Outcomes in Non-Small Cell Lung Cancer. J Natl Compr Canc Netw (2019) 17:574–8. doi: 10.6004/jnccn.2019.5005 31117034

[10] Leighl NB Redman MW Rizvi N Hirsch FR Mack PC Schwartz LH. Phase II Study of Durvalumab Plus Tremelimumab as Therapy for Patients With Previously Treated Anti-PD-1/PD-L1 Resistant Stage IV Squamous Cell Lung Cancer (Lung-MAP Substudy S1400F, Nct03373760). J Immunother Cancer (2021) 9:e002973. doi: 10.1136/jitc-2021-002973 34429332 PMC8386207

[11] Arrieta O Barrón F Ramírez-Tirado LA Zatarain-Barrón ZL Cardona AF Díaz-García D. Efficacy and Safety of Pembrolizumab Plus Docetaxel *vs* Docetaxel Alone in Patients With Previously Treated Advanced Non-Small Cell Lung Cancer: The PROLUNG Phase 2 Randomized Clinical Trial. JAMA Oncol (2020) 6:856–64. doi: 10.1001/jamaoncol.2020.0409 PMC7290397 32271354

[12] Duan C Cheng W Chen Q Li X Zhang J. Pharmacokinetics and Tissue Distribution of Cantharidin After Oral Administration of Aqueous Extracts From Mylabris in Rats. BioMed Chromatogr (2021) 35:e5172. doi: 10.1002/bmc.5172 33982312

[13] Jakovac-Strajn B Brozić D Tavčar-Kalcher G Babič J Trilar T Vengust M. Entomological Surveillance and Cantharidin Concentrations in Mylabris Variabilis and Epicauta Rufidorsum Blister Beetles in Slovenia. Anim (Basel) (2021) 11:220. doi: 10.3390/ani11010220 PMC7830541 33477415

[14] Wang G Dong J Deng L. Overview of Cantharidin and Its Analogues. Curr Med Chem (2018) 25:2034–44. doi: 10.2174/0929867324666170414165253 28413963

[15] Zhou Y Wang M Pan X Dong Z Han L Ju Y. Combination of Triptolide With Sodium Cantharidinate Synergistically Enhances Apoptosis on Hepatoma Cell Line 7721. Zhong Nan Da Xue Xue Bao Yi Xue Ban (2016) 41:911–7. doi: 10.11817/j.issn.1672-7347.2016.09.005 27640789

[16] Zhu M Liu X Zhou C Li J. Effect of Sodium Cantharidinate/Vitamin B6 Injection on Survival, Liver Function, Immune Function, and Quality of Life in Patients With Hepatocellular Carcinoma: Protocol for a Meta-Analysis. Med (Baltimore) (2020) 99:e21952. doi: 10.1097/MD.0000000000021952 PMC7447480 32846865

[17] Shao H Hong G Luo X. Evaluation of Sodium Cantharidinate/Vitamin B6 in the Treatment of Primary Liver Cancer. J Cancer Res Ther (2014) 10:75–8. doi: 10.4103/0973-1482.139770 25207897

[18] Liang F Wang MY Huang WB Li AJ. Effect of Sodium Cantharidinate on the Angiogenesis of Nude Mice With Human Gastric Cancer. Zhong Yao Cai (2011) 34:343–6. 21823448

[19] Chen X Zhou M Fan W Yang M Yang L. Combination of Sodium Cantharidinate With Cisplatin Synergistically Hampers Growth of Cervical Cancer. Drug Des Devel Ther (2021) 15:171–83. doi: 10.2147/DDDT.S282777 PMC7812528 33469269

[20] Liu X Zhang L Thu PM Min W Yang P Li J. Sodium Cantharidinate, a Novel Anti-Pancreatic Cancer Agent That Activates Functional P53. Sci China Life Sci (2020) 64:1295–310. doi: 10.1007/s11427-019-1753-3 33165811

[21] Kong DL Liu Y Wang JY Liu G Zhang ML. Sodium Cantharidinate Suppresses Human Osteosarcoma MG–63 Cell Proliferation and Induces Cell Cycle Arrest by Inhibition of PI3K/AKT Activation. Oncol Rep (2019) 41:1351–8. doi: 10.3892/or.2018.6906 30535442

[22] Wang B Cui J. Treatment of Mid-Late Stage NSCLC Using Sodium Cantharidinate/Vitamin B6/GP Regimen in Clinic. J Cancer Res Ther (2014) 10:C79–81. doi: 10.4103/0973-1482.139771 25207898

[23] Eisenhauer EA Therasse P Bogaerts J Schwartz LH Sargent D Ford R. New Response Evaluation Criteria in Solid Tumours: Revised RECIST Guideline (Version 1.1). Eur J Cancer (2009) 45:228–47. doi: 10.1016/j.ejca.2008.10.026 19097774

[24] Available at: https://db.yaozh.com/linchuangshiyan.

[25] Tao R Sun WY Yu DH Qiu W Yan WQ Ding YH. Sodium Cantharidinate Induces HepG2 Cell Apoptosis Through LC3 Autophagy Pathway. Oncol Rep (2017) 38:1233–9. doi: 10.3892/or.2017.5779 28677738

[26] Fu NW Zhang LS Quan LP. Anti-Tumor Effect and Pharmacology of Sodium Cantharidinate. Chin J Oncol (1980) 2:96–100. doi: 10.2147/DDDT.S282777 7418606

[27] Lu BL Zhang SQ Zhang YH. Anticancer Effect of Sodium Cantharidinate in Mice. Acta Pharm Sin (1980) 2:78–80+130.

[28] Wang GS. Preparation of Sodium Cantharidinate and Determination of Its Structural Formula. Chin Pharm J (1980) 4:40.

[29] Wen SQ Chen Q Hu M. Experimental Study on the Inhibitory Effect of Sodium Cantharidinate on Human Hepatoma HepG2 Cells. Afr J Tradit Complement Altern Med (2013) 11:131–4. doi: 10.4314/ajtcam.v11i1.20 PMC3957254 24653566

[30] Tao R Wang ZF Qiu W He YF Yan WQ Sun WY. Role of S100A3 in Human Hepatocellular Carcinoma and the Anticancer Effect of Sodium Cantharidinate. Exp Ther Med (2017) 13:2812–8. doi: 10.3892/etm.2017.4294 PMC5450779 28588665

[31] Fang DF Wang MY. Experimental Study of Sodium Cantharidinate Inhibiting the Growth of Liver Cancer Cells. Liaoning J Traditional Chin Med (2007) 06:845–7.

[32] Feng L Huang WL Huang WB. The Effect of Sodium Cantharidinate on the Proliferation, Apoptosis and Caspase-3 Expression of Subcutaneously Transplanted Tumor Cells in H_ (22) Tumor-Bearing Mice. J Baotou Med Coll (2018) 34:81–83+89.

[33] Chen KL Yuan HC Li SL. The Effect of Sodium Cantharidinate on the Metabolism of Ibrutinib in Rats. Chin J Clin Pharmacol (2019) 35:161–4.

[34] Zhu WZ Tong Z Xu T. Analysis of the Efficacy of Sodium Cantharidinate and Vitamin B_6 Combined With Chemotherapy on Lung Cancer. World Chin Med (2020) 15:1607–10.

[35] Xiao Z Wang C Tan Z. Clinical Efficacy and Safety of Sodium Cantharidinate Plus Chemotherapy in Non-Small-Cell Lung Cancer: A Systematic Review and Meta-Analysis of 38 Randomized Controlled Trials. J Clin Pharm Ther (2019) 44:23–38. doi: 10.1111/jcpt.12761 30229971

[36] Li Z Liu Z Wu Y. Efficacy and Safety of Apatinib Alone or Apatinib Plus Paclitaxel/Docetaxel Versus Paclitaxel/Docetaxel in the Treatment of Advanced Non-Small Cell Lung Cancer: A Meta-Analysis. Thorac Cancer (2021) 12:2838–48. doi: 10.1111/1759-7714.14131 PMC8563161 34622571

[37] Matsumoto K Tamiya A Inagaki Y Taniguchi Y Matsuda Y Kawachi H. Efficacy and Safety of Ramucirumab Plus Docetaxel in Older Patients With Advanced Non-Small Cell Lung Cancer: A Multicenter Retrospective Cohort Study. J Geriatr Oncol (2021) 30:S1879–4068(21) 00205-8. doi: 10.1016/j.ijrobp.2021.04.017 34602370

[38] Maldonado F Gonzalez-Ling A Oñate-Ocaña LF Cabrera-Miranda LA Zatarain-Barrón ZL Turcott JG. Prophylactic Cranial Irradiation in Patients With High-Risk Metastatic Non-Small Cell Lung Cancer: Quality of Life and Neurocognitive Analysis of a Randomized Phase II Study. Int J Radiat Oncol Biol Phys (2021) 111:81–92. doi: 10.1016/j.ijrobp.2021.04.017 33915217

[39] World Medical Association. World Medical Association Declaration of Helsinki: Ethical Principles for Medical Research Involving Human Subjects. JAMA (2013) 310:2191–4. doi: 10.1001/jama.2013.281053 24141714

[40] Basch E Becker C Rogak LJ Schrag D Reeve BB Spears P. Composite Grading Algorithm for the National Cancer Institute's Patient-Reported Outcomes Version of the Common Terminology Criteria for Adverse Events (PRO-CTCAE). Clin Trials (2021) 18:104–14. doi: 10.1177/1740774520975120 PMC7878323 33258687

[41] Liu YL Yang ZF Li Z. Observation on the Efficacy of Sodium Cantharidinate and Vitamin B6 Injection in the Treatment of Malignant Pleural Effusion and Ascites. Chin J Cancer Prev Treat (2006) 19:1519–20.

