# Supplementary figures and images for: Efficacy and Safety of Docetaxel and Sodium Cantharidinate Combination vs. Either Agent Alone as Second-Line Treatment for Advanced/Metastatic NSCLC With Wild-Type or Unknown EGFR Status: An Open-Label, Randomized Controlled, Prospective, Multi-Center Phase III Trial (Cando-L1)
Source: Front Oncol. 2021 Dec 14;11:769037. doi: 10.3389/fonc.2021.769037 (PMC8715707; doi:10.3389/fonc.2021.769037)

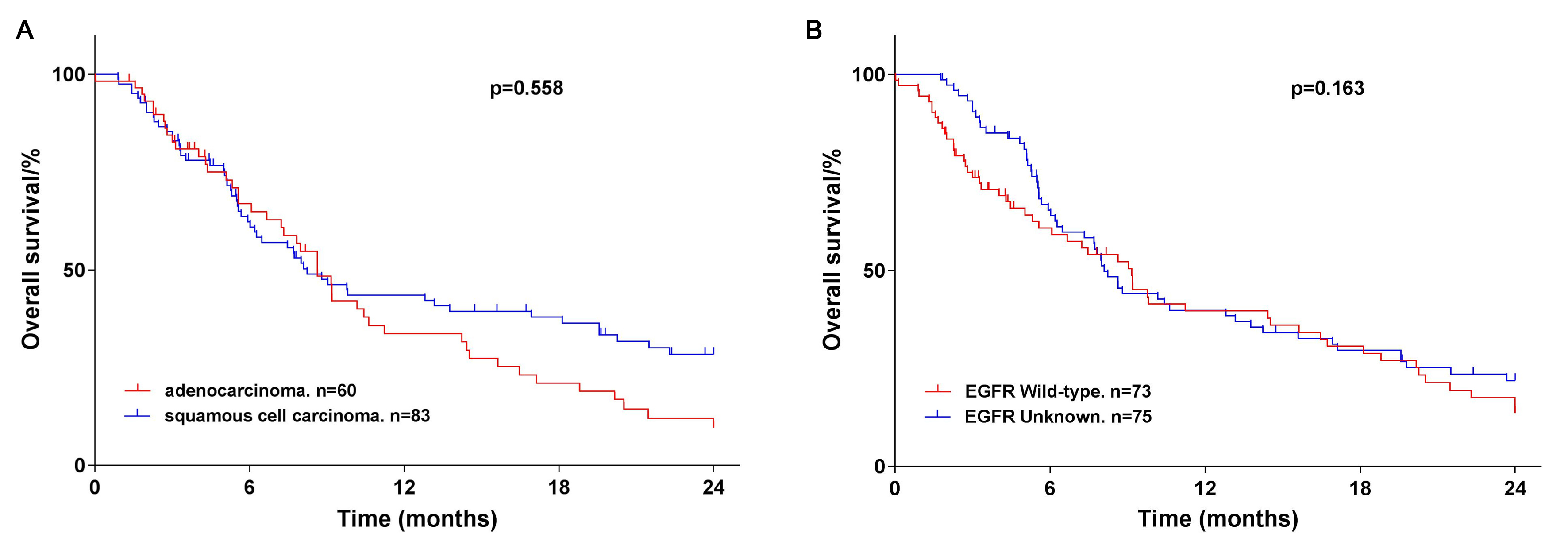

Supplement: Supplementary Figure 1 — Kaplan-Meier curves for overall survival (OS) among (A) histological type and (B) EGFR status. [file Image_1.tif]
