# Supplementary material for: Efficacy and Safety of Docetaxel and Sodium Cantharidinate Combination vs. Either Agent Alone as Second-Line Treatment for Advanced/Metastatic NSCLC With Wild-Type or Unknown EGFR Status: An Open-Label, Randomized Controlled, Prospective, Multi-Center Phase III Trial (Cando-L1)
Source: Front Oncol. 2021 Dec 14;11:769037. doi: 10.3389/fonc.2021.769037 (PMC8715707; doi:10.3389/fonc.2021.769037)
Supplement: Supplementary file 2 [file DataSheet_1.docx]

**Supplementary Table**

**Supplementary Table 1**. Included Patients From Each Institution.

| Institution Name | PI | No. of patients |
| --- | --- | --- |
| S01 Second Xiangya Hospital of Central South University | Chunhong Hu | 42 |
| S02 The First Affiliated Hospital of South China University | Youhua Wu | 7 |
| S03 The First People's Hospital of Yueyang | Jie Weng | 10 |
| S04 The Central Hospital of Yiyang | Bing Zhang | 4 |
| S05 Xiangya Hospital of Central South University | Shan Zeng | 6 |
| S06 The Central Hospital of Zhuzhou | Bo Qiu | 5 |
| S07 Hunan Cancer Hospital | Lin Wu | 45 |
| S08 The Central Hospital of Xiangtan | Tiegang Tang | 6 |
| S09 Third Xiangya Hospital of Central South University | Peiguo Cao | 5 |
| S11 Hunan Provincial People's Hospital | Huaxin Duan | 4 |
| S12 The Central Hospital of Shaoyang, Shaoyang | Hui Zhang | 11 |
| S14 Peking Union Medical College Hospital | Li Zhang | 5 |
| S15 Chinese PLA General Hospital | Dong Zhang | 1 |

**Supplementary Table 2.** Patients’ baseline demographic and clinical characteristics (N=148).

| **Characteristic** |  |
| --- | --- |
| Median age at enrollment– years (IQR), n=148 | 51(44-61) |
| Male sex– no. (%) | 124(83.78) |
| Smoking– no. (%) | 104(70.27) |
| Median ECGO at enrollment– years (IQR), n=148 | 1(1-1) |
| Median Charlson Comorbidity Index (CCI) at enrollment, points. (IQR), n=148 | 8(7-8) |
| Cancer stage IV– no. (%) | 148(100) |
| Histological type– no. (%) |  |
| Adenocarcinoma | 60(40.54) |
| Squamous cell carcinoma | 83(56.08) |
| Others | 5(3.38) |
| EGFR status– no. (%) |  |
| Wild-type | 73(49.32) |
| Unknown | 75(50.68) |
| Median Treatment cycle, (IQR) | 2(1-4) |

*IQR: denotes interquartile range, SD: Standard Deviation, CCI: Charlson Comorbidity Index.

**Supplementary Table 3.** Most frequent adverse events (N=148).

| **AEs Category** | **Total(N=148)** | | | **SCA(n=50)** | | **DOX(n=48)** | | **CON(n=50)** | |
| --- | --- | --- | --- | --- | --- | --- | --- | --- | --- |
|  | | **All** | **Grade ≥ 3** | **All** | **Grade ≥ 3** | **All** | **Grade ≥ 3** | **All** | **Grade ≥ 3** |
| **Hematologic AEs** | |  |  |  |  |  |  |  |  |
| Neutropenia decreased | | 58(39) | 17(12) | 7(14) | 1(2) | 25(52) | 7(15) | 26(52) | 9(18) |
| Hemoglobin decreased | | 40(27) | 5(3) | 8(16) | 1(2) | 15(31) | 1(2) | 17(34) | 3(6) |
| Platelet count decreased | | 15(10) | 0(0) | 3(6) | 0(0) | 6(13) | 0(0) | 6(12) | 0(0) |
| **Nonhematologic AEs** | |  |  |  |  |  |  |  |  |
| Asthenia/fatigue/Lethargy | | 78(53) | 5(3) | 23(46) | 2(4) | 27(56) | 2(4) | 28(56) | 1(2) |
| Nausea/Vomiting | | 70(47) | 3(3) | 12(24) | 1(1) | 28(58) | 2(4) | 30(60) | 0(0) |
| Increase in ALT/AST | | 65(44) | 2(2) | 18(36) | 0(0) | 22(46) | 0(0) | 25(50) | 2(4) |
| Constipation | | 58(39) | 0(0) | 8(16) | 0(0) | 24(50) | 0(0) | 26(52) | 0(0) |
| Abdominal pain/Diarrhea | | 30(20) | 0(0) | 8(16) | 0(0) | 12(25) | 0(0) | 10(20) | 0(0) |
| Increase in creatinine | | 25(17) | 0(0) | 6(12) | 0(0) | 9(19) | 0(0) | 10(20) | 0(0) |
| Muscle soreness | | 19(13) | 0(0) | 5(10) | 0(0) | 5(10) | 0(0) | 9(18) | 0(0) |
| Allergy/Rash | | 10(7) | 0(0) | 2(4) | 0(0) | 3(6) | 0(0) | 5(10) | 0(0) |

* AEs: adverse events, ALT: Alanine aminotransferase, AST: Aspartate aminotransferase.
